# Clinical phenotypes of type 2 diabetes and their association with microvascular complications in primary care: a cluster analysis

**DOI:** 10.1186/s12875-026-03458-4

**Published:** 2026-07-06

**Authors:** Alcibíades Segundo Díaz Vera, José Abellán Alemán, Miladi del Socorro Golac Rabanal, Cédric Marco Detchart, Isabel Arrechea Irigoyen, Manuela García Sánchez, Daime Pérez Feíto, Virginia Íñiguez García, Roberto Dair García de la Rosa, Rosa Eva Perez–Siguas, Hernán Hugo Matta–Solís, Eduardo Percy Matta–Solís, María Lourdes Azcona Cortés, Julieth Paola Sulaiman Rojas, Yohanna Elizabeth Barría Jujihara, Julienne Ntumba Mumba, Martín Andrés Astudillo Astudillo, Carlos José Zhuzhingo Vásquez, Javier Nieto Iglesias, Luis Alberto Díaz Vera, Gonzalo Luis Alonso Salinas

**Affiliations:** 1 Institute for Health Research of Navarra (IdiSNA), Pamplona, Spain; 2 Department of Health Sciences (Preventive Medicine and Public Health), Public University of Navarra, Pamplona, Spain; 3 Primary Care, Servicio Navarro de Salud–Osasunbidea, Pamplona, Spain; 4 Department of Cardiology, Hospital Universitario de Navarra, Pamplona, Spain; 5 Department of Health Sciences, Public University of Navarra (UPNA), Pamplona, Spain; 6 Navarrabiomed,Miguel Servet Foundation, Pamplona, Spain; 7 Chair of Cardiovascular Risk, Catholic University of Murcia (UCAM), Murcia, Spain; 8 Department of Statistics, Computer Science and Mathematics, Public University of Navarre (UPNA), Pamplona, Spain; 9 Hospital Universitario San Pedro, Logroño, Spain; 10 Hospital General Universitario de Ciudad Real, Servicio de Salud de Castilla-La Mancha (SESCAM), Ciudad Real, Spain; 11 Hospital Nacional Edgardo Rebagliati Martins, Lima, Peru; 12 International Doctoral School, Catholic University of Murcia (UCAM), Murcia, Spain; 13 Escuela de Posgrado Newman S.A.C. Unidad de Posgrado Ciencias Médicas de la Salud, Lima, Peru; 14 Instituto Peruano de Salud Familiar, Lima, Peru; 15 Escuela de Postgrado, Universidad María Auxiliadora, Lima, Peru

**Keywords:** Type 2 diabetes mellitus, Cluster analysis, Clinical phenotypes, Diabetic retinopathy, Diabetic nephropathy, Primary care, Microvascular complications, Cross-sectional study

## Abstract

**Background:**

Type 2 diabetes mellitus is a heterogeneous condition associated with a substantial burden of microvascular complications. Conventional assessment based on isolated clinical variables may not fully capture this heterogeneity. Cluster analysis offers a potential strategy to identify clinically relevant diabetes phenotypes using routinely collected primary care variables.

**Objective:**

To identify clinical phenotypes of type 2 diabetes in a primary care population using cluster analysis and to examine their association with prevalent diabetic retinopathy and nephropathy.

**Methods:**

A cross-sectional study was conducted using routinely collected data from primary care centres in the Cantabrian Health Service. Centres were randomly selected using probability proportional to size sampling. People with type 2 diabetes mellitus were identified from clinical records, and those with missing key variables were excluded. K-means clustering was applied using routinely collected clinical variables, including body mass index, systolic and diastolic blood pressure, HbA1c, age, and years since diagnosis. Associations between clinical phenotypes and prevalent microvascular complications were assessed using logistic regression models adjusted for age, sex, and years since diagnosis. Model discrimination and calibration were evaluated using the area under the receiver operating characteristic curve (AUC), Hosmer–Lemeshow test, Nagelkerke R², and Brier score.

**Results:**

Of 742 initially identified individuals, 680 were included in the final analytical sample.

Phenotype assignment was possible for 674 individuals, who were classified into three clinically interpretable diabetes phenotypes: controlled (*n* = 492), metabolic (*n* = 146), and hypertensive (*n* = 36). The prevalence of diabetic retinopathy was 20.5%, 58.2%, and 27.8%, respectively, while the prevalence of diabetic nephropathy was 25.9%, 51.1%, and 27.8%. After adjustment, the metabolic phenotype was associated with higher odds of prevalent retinopathy (OR 4.69, 95% CI 2.99–7.36; *p* < 0.001) and prevalent nephropathy (OR 2.52, 95% CI 1.67–3.79; *p* < 0.001) compared with the controlled phenotype. The hypertensive phenotype was associated with prevalent retinopathy (OR 2.43, 95% CI 1.03–5.69; *p* = 0.042), but not with prevalent nephropathy (OR 1.23, 95% CI 0.56–2.68; *p* = 0.611). The retinopathy model showed good discrimination (AUC 0.82, 95% CI 0.78–0.86), whereas the nephropathy model showed lower discrimination (AUC 0.67, 95% CI 0.63–0.72). Sensitivity analyses yielded consistent results.

**Conclusions:**

Three clinically interpretable diabetes phenotypes were identified in a real-world primary care population and were associated with different patterns of prevalent microvascular complications. The metabolic phenotype showed the highest prevalence and higher adjusted odds of both retinopathy and nephropathy, while the hypertensive phenotype showed a more selective association with retinopathy. These findings suggest that routinely collected primary care data can be used to describe clinically meaningful patterns of co-occurrence between type 2 diabetes phenotypes and microvascular complications. Given the cross-sectional design, these findings should not be interpreted as predictive or causal, and longitudinal studies and external validation are required before these phenotypes can be considered for clinical implementation.

**Supplementary Information:**

The online version contains supplementary material available at https://doi.org/10.1186/s12875-026-03458-4.

## Introduction

Type 2 diabetes mellitus is a highly prevalent chronic condition and a major contributor to morbidity and healthcare use worldwide, affecting more than 500 million adults globally [1]. Its clinical course is strongly influenced by the development of microvascular complications, particularly diabetic retinopathy and diabetic nephropathy, which are associated with visual impairment, kidney function decline, reduced quality of life, and increased healthcare burden [2, 3]. In primary care, where most people with type 2 diabetes are routinely followed, identification of clinical profiles associated with a higher burden of complications remains a major clinical priority, particularly given the persistence of HbA1c levels above target and therapeutic inertia in routine clinical practice [4, 5].

Traditionally, clinical assessment in type 2 diabetes has relied on individual factors such as glycaemic status, blood pressure, obesity, and disease duration. Although these variables are clearly relevant, this conventional approach may not fully capture the marked heterogeneity of people with type 2 diabetes [6, 7]. Individuals with apparently similar diagnoses may differ substantially in their metabolic characteristics, blood pressure profile, and burden of complications. In this context, clinical phenotype-based approaches have gained increasing interest as a means of better characterising disease heterogeneity.

Although several validated tools exist for the early detection of prediabetes and type 2 diabetes, integrated approaches to identify clinical profiles associated with microvascular complications, including eye and kidney disease, appear to be less established in routine primary care.

Systematic evidence suggests that most stratification approaches in type 2 diabetes are based on predefined expert-driven criteria, while data-driven methods remain underutilised, limiting the identification of clinically meaningful subgroups [6]. Cluster analysis, an unsupervised statistical learning approach, offers a data-driven strategy to identify subgroups of people with shared clinical characteristics without imposing predefined categories [8].

In diabetes research, clustering approaches have increasingly been used to define clinically meaningful phenotypes associated with differences in clinical characteristics, complication burden, and patterns of care. A landmark data-driven study identified five distinct subgroups of adult-onset diabetes with significantly different clinical characteristics, disease progression, and complication profiles, highlighting the potential of refined classification systems to better describe diabetes heterogeneity [8]. Studies validating these data-driven classifications have demonstrated that distinct diabetes subgroups are associated with different patterns of microvascular complications [9]. Similarly, clustering analyses have identified subgroups of people with type 2 diabetes showing different profiles of chronic complications, including diabetic retinopathy and nephropathy [10]. Importantly, real-world studies conducted in primary care settings have shown that clustering based on routinely collected clinical data can identify clinically relevant subgroups with distinct clinical characteristics and complication patterns [11]. In addition, clustering approaches have been shown to identify subgroups with distinct pathophysiological profiles and differential responses to treatment, highlighting their potential relevance for better describing heterogeneity in type 2 diabetes care [12].

However, much of the existing literature has been developed in specialised or research settings, often relying on complex biomarkers or less readily transferable analytical frameworks, which may limit implementation in routine clinical practice. Despite these advances, there remains a lack of clinically applicable and clinically interpretable phenotype-based approaches that can be readily implemented using routinely collected primary care data.

From a clinical perspective, this issue is particularly relevant because major determinants of microvascular complications, such as hyperglycaemia and hypertension, may cluster differently across people, potentially leading to distinct patterns of co-occurring complications despite a shared diagnosis of type 2 diabetes. In this study, the terms “metabolic phenotype” and “hypertensive phenotype” are used descriptively to summarise the dominant clinical characteristics of the clusters identified in the dataset, rather than to imply fixed, mutually exclusive, or externally validated diabetes subtypes.

Therefore, there is a need for clinically interpretable and easily implementable phenotyping strategies based exclusively on variables routinely available in primary care.

The aim of this study was to identify clinically meaningful phenotypes of type 2 diabetes in a primary care population using cluster analysis based on routinely available variables, and to examine their cross-sectional association with prevalent diabetic retinopathy and nephropathy. We additionally assessed model discrimination, calibration, and robustness as descriptive indicators of how phenotype-based models distinguished individuals with and without prevalent microvascular complications in routine primary care data.

## Methods

### Study design and participants

A cross-sectional study was conducted using routinely collected data from primary care centres within the Cantabrian Health Service. The study was based on a secondary analysis of a previously established population-based cohort of people with type 2 diabetes, whose design and data collection methods have been described elsewhere [13].

The original cohort was derived using a multistage random sampling strategy with probability proportional to size. Of the 42 primary care centres in Cantabria, 13 centres (approximately one-third) were randomly selected. Within each participating centre, a further random selection of physician patient lists was performed to obtain a representative sample of people with type 2 diabetes, ensuring representativeness of the primary care diabetes population in Cantabria.

**Fig. 1:**
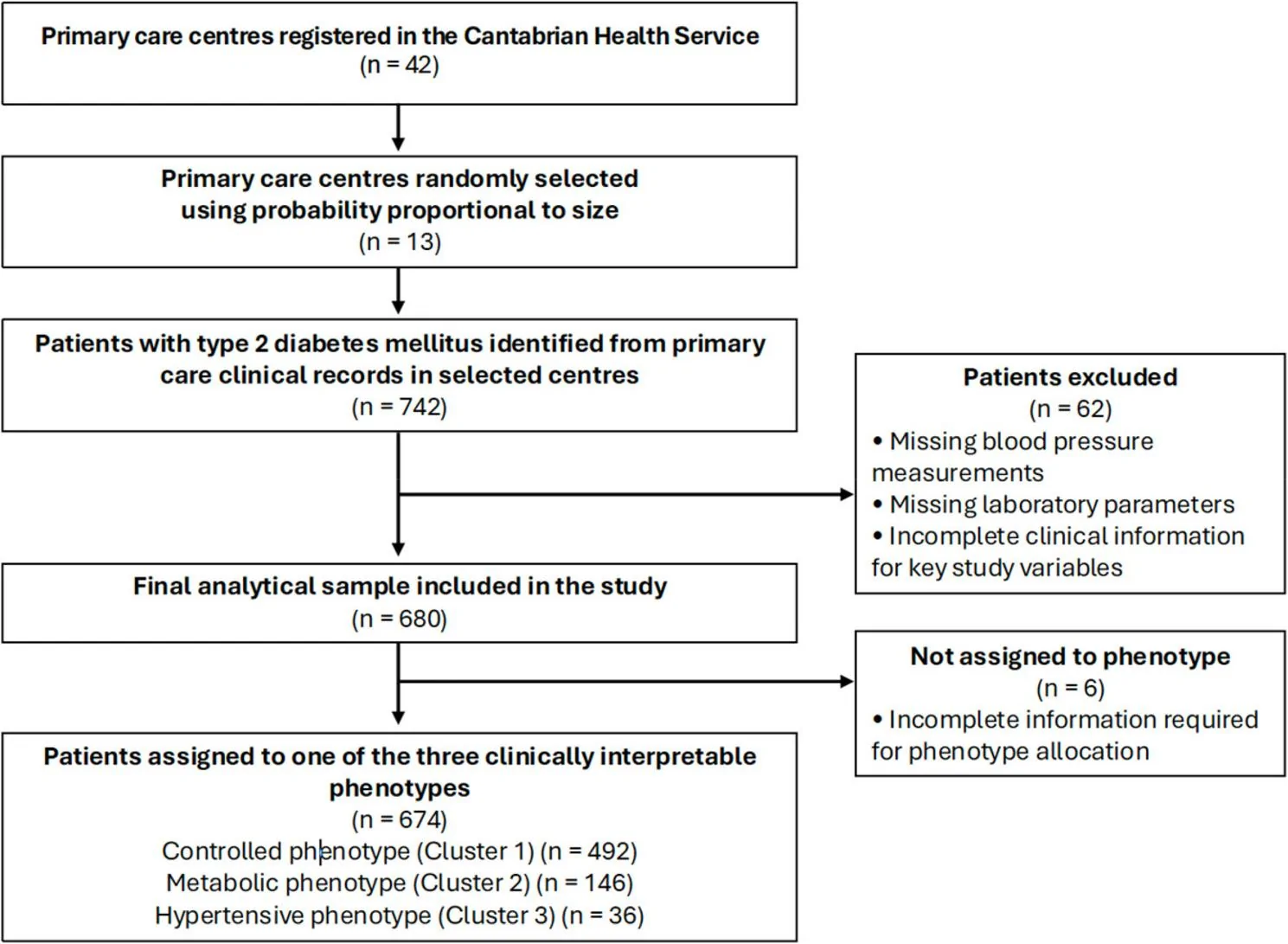
Flow diagram of patient selection, inclusion, and phenotype assignment in the study. Primary care centres were randomly selected from the Cantabrian Health Service using probability proportional to size sampling. Patients with type 2 diabetes mellitus were identified from primary care clinical records in the selected centres. A total of 742 patients were initially identified, of whom 62 were excluded because of missing blood pressure measurements, incomplete laboratory parameters, or incomplete clinical information for key study variables. The final analytical sample comprised 680 patients. Phenotype assignment was possible for 674 patients with complete information for the classification criteria; 6 patients were not assigned to one of the three clinical phenotypes

People with type 2 diabetes were identified from electronic health records in the participating centres. Type 2 diabetes was defined as a previous diagnosis recorded in primary care clinical records or the use of antidiabetic medication. Data collection for the original cohort was conducted between January 2014 and December 2015. A total of 742 individuals were initially identified. After exclusion of 62 individuals due to missing blood pressure measurements, incomplete laboratory data, or missing key study variables, the final analytical sample comprised 680 individuals with sufficient data for inclusion in at least one study analysis. Phenotype assignment was subsequently performed among individuals with complete information for the variables required for classification. All phenotype-based analyses were restricted to the 674 individuals assigned to one of the three clinical phenotypes; analyses of each outcome were further restricted to individuals with available outcome data. The availability of key variables in the final analytical sample is reported in Supplementary Table S2. A detailed flow diagram of patient selection, inclusion, and phenotype assignment is provided in Fig. 1.

### Variables

Descriptive variables included age, sex, and duration of diabetes, as summarised in Table 1. Variables used for cluster derivation were body mass index (BMI), systolic blood pressure, diastolic blood pressure, HbA1c, age, and duration of diabetes, consistent with the cluster profiles presented in Table 3; Fig. 3.

**Table 1:** Baseline characteristics of patients assigned to clinical phenotypes

Variable	Controlled phenotype (Cluster 1) (n=492)	Metabolic phenotype (Cluster 2) (n=146)	Hypertensive phenotype (Cluster 3) (n=36)	p-value
Age (years), mean ± SD	70.3 ± 12.9	68.9 ± 14.7	65.6 ± 11.3	0.026
Male sex, n (%)	251 (51.0%)	74 (50.7%)	26 (72.2%)	0.045
Years since diagnosis, mean ± SD	9.5 ± 4.1	12.0 ± 5.7	7.9 ± 3.8	<0.001

Data are presented as mean ± standard deviation (SD) for continuous variables and as counts and percentages (n, %) for categorical variables. This table includes patients assigned to one of the three clinical phenotypes (n = 674). p-values were calculated using analysis of variance (ANOVA) for continuous variables and the χ² test for categorical variables

Clinical, sociodemographic, anthropometric, and laboratory data were obtained from electronic health records, direct clinical assessment, and structured interviews conducted during study visits. Anthropometric measurements, including BMI, were obtained during clinical assessment following standardised procedures. Laboratory parameters, including HbA1c, were obtained from routine clinical care and corresponded to repeated determinations during the study period, including study entry, 6 months, and 12 months, as described in the original cohort. For clustering, HbA1c was represented by the mean of the available determinations during the study assessment window, consistent with the operational definition used in the original cohort. Blood pressure measurements were obtained during routine primary care visits using validated automated devices and were based on the mean of three measurements obtained across successive visits, in accordance with standard clinical practice.

BMI was analysed as a continuous variable. HbA1c and blood pressure measurements were dichotomised only to facilitate descriptive interpretation of the resulting phenotypes. HbA1c above target was defined as HbA1c ≥ 7.0%; elevated systolic blood pressure was defined as systolic blood pressure ≥ 140 mmHg; and elevated diastolic blood pressure was defined as diastolic blood pressure ≥ 90 mmHg. Continuous values were used for clustering, whereas dichotomised thresholds were applied only for descriptive interpretation of the resulting phenotypes.

The primary outcomes were prevalent diabetic retinopathy and prevalent diabetic nephropathy, both analysed as binary outcomes. Diabetic retinopathy was assessed using the most recent available retinographic examination from the previous year; when no retinographic examination was available, retinography was requested at the time of study inclusion. Diabetic nephropathy was ascertained from renal assessment data available in the original cohort, including urinary albumin-to-creatinine ratio and estimated glomerular filtration rate, as recorded in the clinical dataset. Prevalent diabetic nephropathy was defined according to the renal variables available in the dataset, based on urinary albumin-to-creatinine ratio ≥ 30 mg/g and/or estimated glomerular filtration rate < 60 mL/min/1.73 m². Outcomes were ascertained from electronic health records and routine clinical assessments during the study period. All clinical variables and outcomes were considered as part of the same cross-sectional assessment window, and no temporal sequence between phenotype assignment and complications was assumed. Therefore, the analysis evaluates cross-sectional associations and patterns of co-occurrence between clinical phenotypes and prevalent microvascular complications, rather than incident outcomes or prediction over follow-up.

### Cluster analysis

K-means clustering has been widely applied in diabetes research to identify clinically meaningful subgroups based on routinely available metabolic and clinical variables, supporting its use as a data-driven approach for clinical phenotyping [8, 10, 11].

K-means clustering was applied to identify clinical phenotypes in people with type 2 diabetes. Variables included in the clustering process were standardised using z-score transformation prior to analysis to ensure comparability across scales. A three-cluster solution was selected based on statistical adequacy and clinical relevance. Although the silhouette coefficient was slightly higher for the two-cluster solution, the three-cluster model provided greater clinical interpretability and more meaningful phenotypic differentiation while maintaining acceptable cluster separation according to silhouette analysis (Supplementary Figure S1) [14]. Individuals were grouped according to BMI, systolic blood pressure, diastolic blood pressure, HbA1c, age, and duration of diabetes.

After cluster identification, the resulting groups were clinically characterised according to their predominant features and labelled post hoc as controlled, metabolic, or hypertensive phenotypes. The controlled phenotype was defined by HbA1c within target range and the absence of diastolic hypertension; the metabolic phenotype by HbA1c above target, higher BMI, and longer disease duration; and the hypertensive phenotype by predominant elevation of systolic and diastolic blood pressure. The label “controlled phenotype” was used descriptively to indicate HbA1c within target range and absence of diastolic hypertension, not as a value judgement on individual disease management. The final distribution was: controlled (*n* = 492), metabolic (*n* = 146), and hypertensive (*n* = 36).

### Principal component analysis and visualisation

**Fig. 2:**
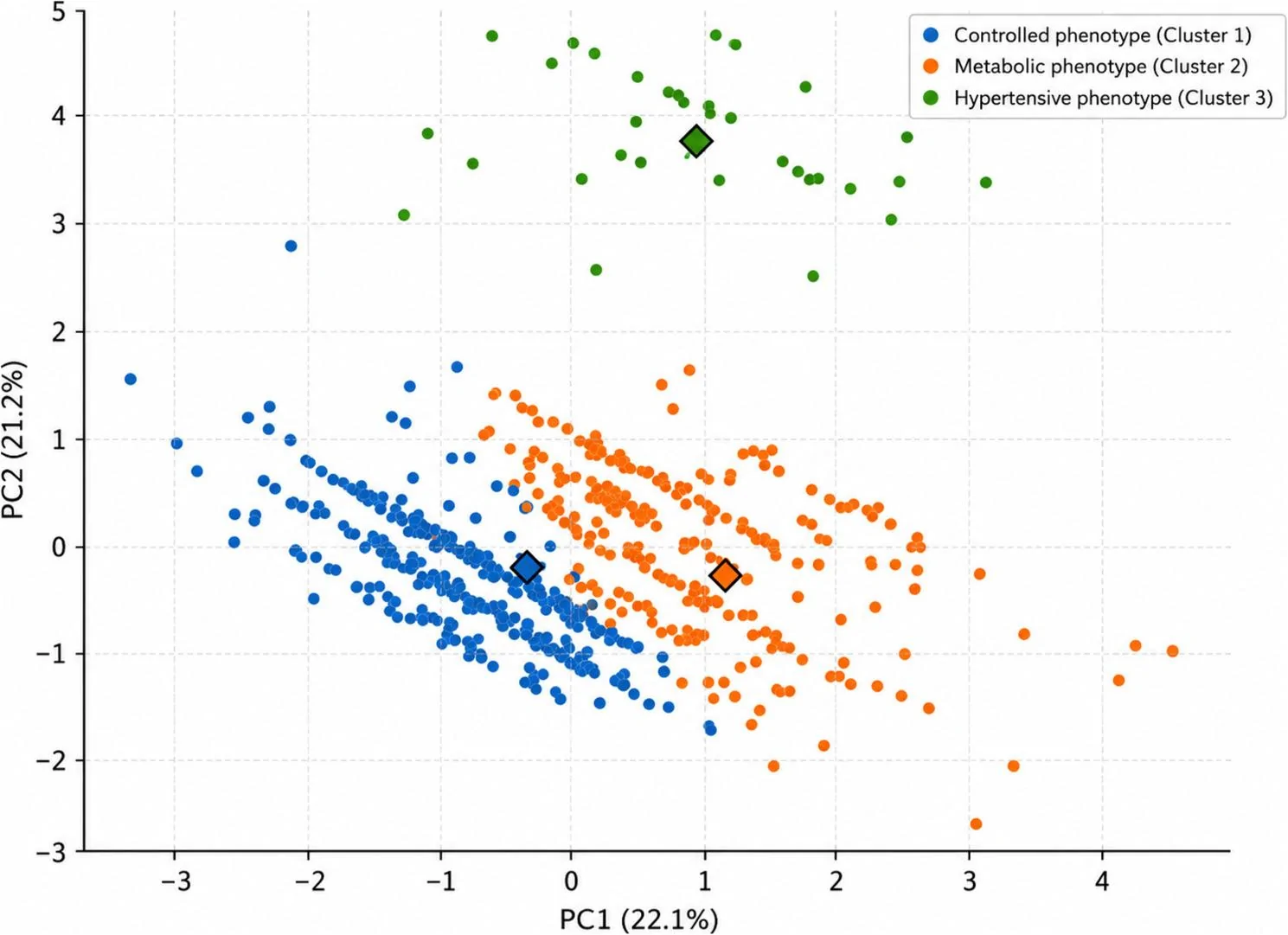
Principal component analysis plot of the identified clinical phenotypes. Principal component analysis (PCA) was used solely for post-hoc two-dimensional visualisation of the identified clinical phenotypes and did not inform phenotype assignment. Phenotype assignment was based on the classification variables described in the Methods. The hypertensive phenotype (Cluster 3) showed visual separation from the remaining phenotypes, whereas partial overlap was observed between the controlled and metabolic phenotypes, suggesting a gradual clinical continuum between these groups

Principal component analysis (PCA) was used solely for post-hoc two-dimensional visualisation of the identified clinical phenotypes and did not inform phenotype assignment. The first two principal components were retained for visualisation purposes only (Fig. 2).

**Fig. 3:**
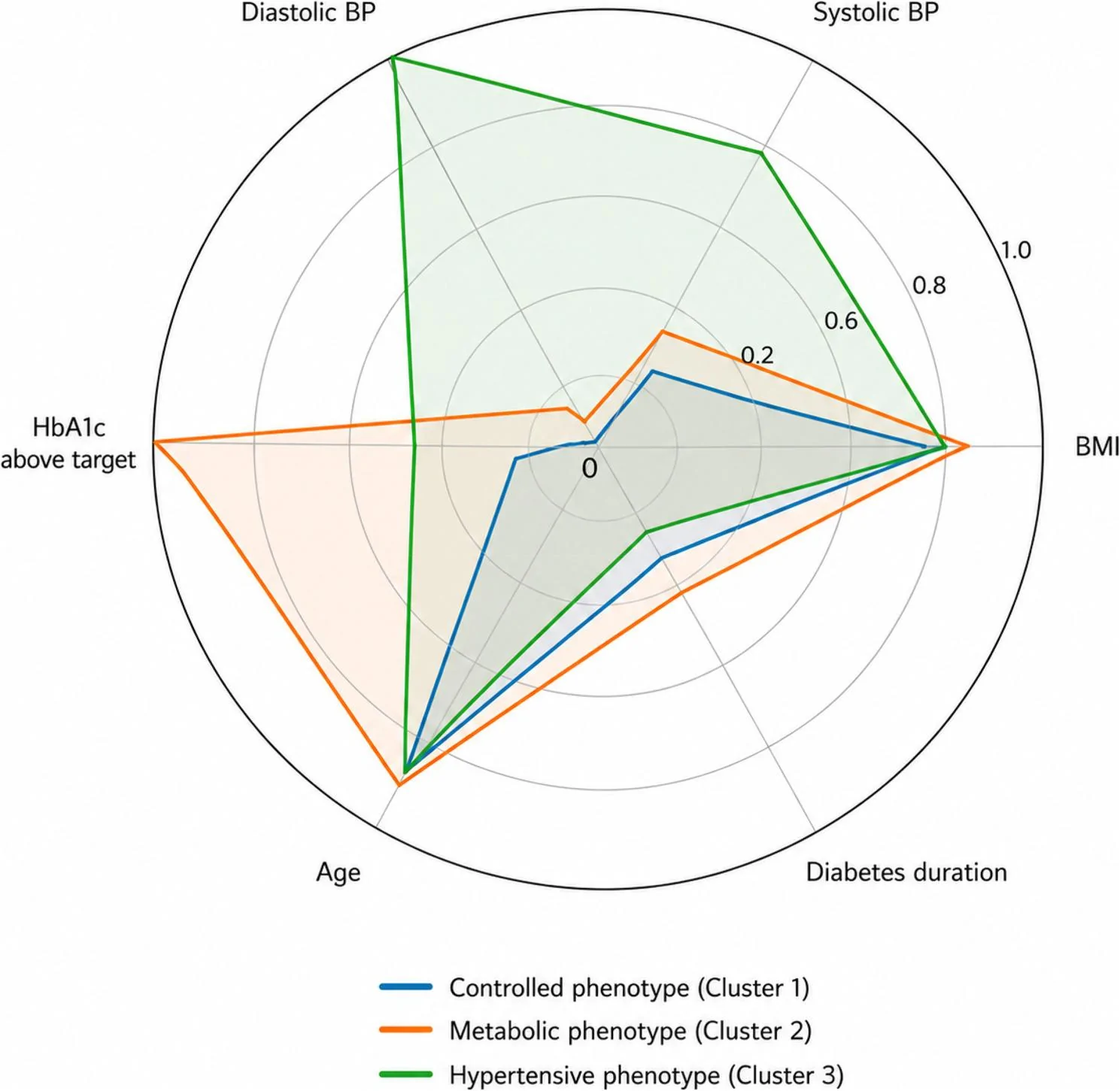
Radar plot of the clinical profiles of the identified phenotypes. Radar plot illustrating the standardised clinical profiles of the three identified phenotypes. Variables were normalised to a 0–1 scale to facilitate comparison across different units. The metabolic phenotype was characterised by HbA1c above target and longer duration of diabetes, whereas the hypertensive phenotype showed predominant systolic and diastolic blood pressure elevation

A radar plot was constructed to illustrate the standardised profiles of the identified phenotypes. Variables were normalised to a 0–1 scale to facilitate comparison across variables with different measurement scales (Fig. 3).

### Statistical analysis

Continuous variables were presented as mean ± standard deviation (SD), and categorical variables as frequencies and percentages. Comparisons across phenotypes were performed using analysis of variance (ANOVA) for continuous variables and the χ² test for categorical variables (Tables 1, 2 and 3).

**Table 2:** Prevalence of microvascular complications by clinical phenotype

Outcome	Controlled phenotype (Cluster 1)	Metabolic phenotype (Cluster 2)	Hypertensive phenotype (Cluster 3)	p-value
Diabetic retinopathy, n (%)	101/492 (20.5%)	85/146 (58.2%)	10/36 (27.8%)	<0.001
Diabetic nephropathy, n (%)	126/486 (25.9%)	72/141 (51.1%)	10/36 (27.8%)	<0.001

Categorical variables are presented as counts and percentages, using the number of patients with available data for each outcome as the denominator

p-values were calculated using the χ² test. Comparisons were performed across the three phenotypes

**Table 3:** Clinical characteristics of patients assigned to clinical phenotypes

Variable	Controlled phenotype (Cluster 1) (n=492)	Metabolic phenotype (Cluster 2) (n=146)	Hypertensive phenotype (Cluster 3) (n=36)	p-value
Age (years), mean ± SD	70.3 ± 12.9	68.9 ± 14.7	65.6 ± 11.3	0.026
Years since diagnosis, mean ± SD	9.5 ± 4.1	12.0 ± 5.7	7.9 ± 3.8	<0.001
BMI (kg/m²), mean ± SD	27.9 ± 4.9	32.6 ± 5.2	31.3 ± 5.3	0.004
Elevated HbA1c, n (%)	0 (0%)	146 (100%)	11 (30.6%)	<0.001
Elevated systolic blood pressure, n (%)	138 (28.0%)	45 (30.8%)	30 (83.3%)	<0.001
Elevated diastolic blood pressure, n (%)	0 (0%)	0 (0%)	36 (100%)	<0.001

Data are presented as mean ± standard deviation (SD) for continuous variables and as counts and percentages (n, %) for categorical variables. This table includes patients assigned to one of the three clinical phenotypes (n = 674). BMI was analysed as a continuous variable. Elevated HbA1c was defined as HbA1c ≥7.0%; elevated systolic blood pressure was defined as systolic blood pressure ≥140 mmHg; and elevated diastolic blood pressure was defined as diastolic blood pressure ≥90 mmHg. These dichotomised variables were used to facilitate the descriptive interpretation of the resulting phenotypes. p-values were calculated using analysis of variance (ANOVA) for continuous variables and the χ² test for categorical variables. Variables related to glycaemic status and blood pressure were included in the classification process and therefore reflect defining characteristics of the identified phenotypes

**Fig. 4:**
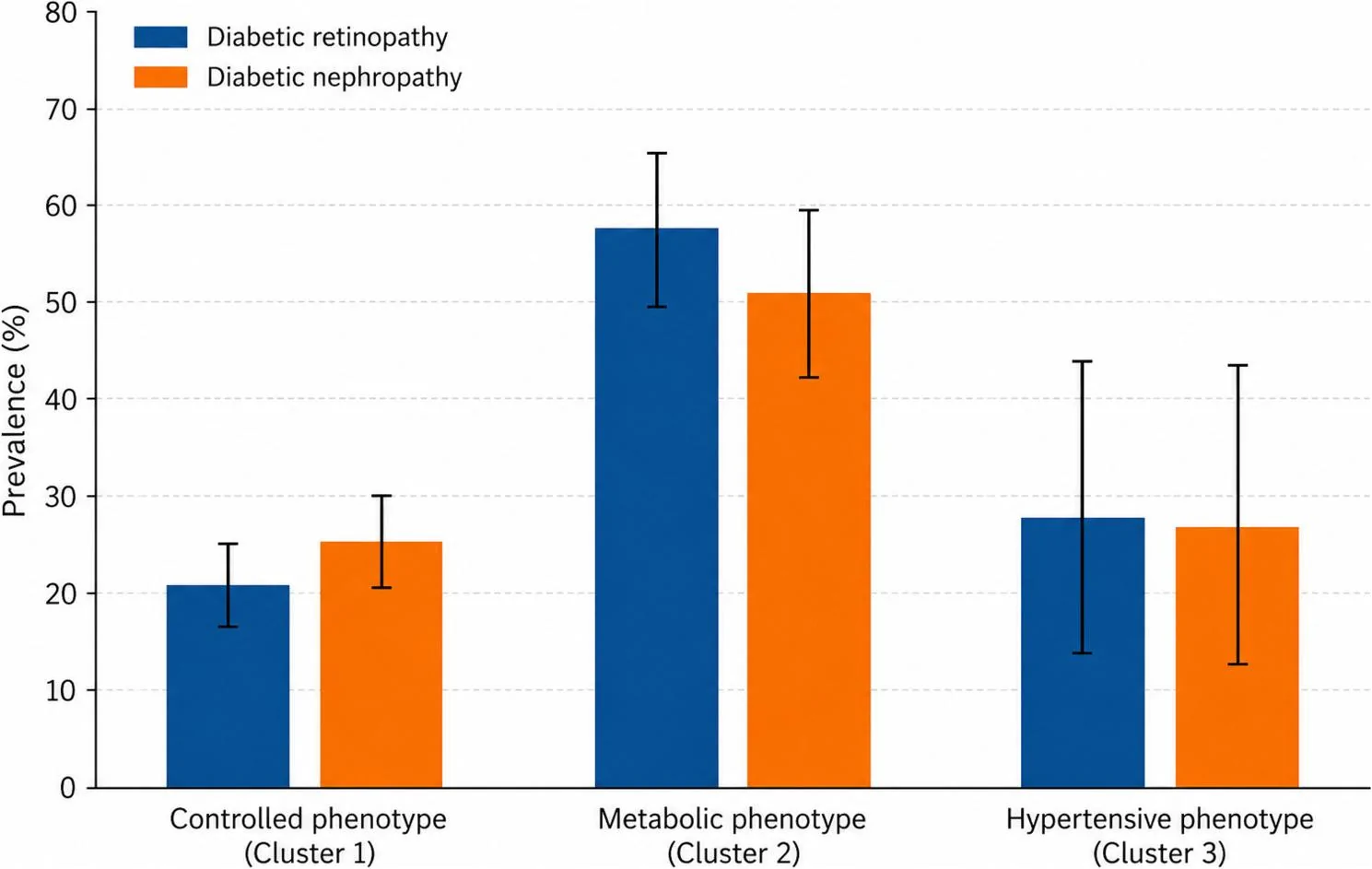
Microvascular complications across clinical phenotypes. Bar chart showing the prevalence of diabetic retinopathy and diabetic nephropathy across the identified clinical phenotypes. Error bars represent 95% confidence intervals. Percentages were calculated using the number of patients with available data for each outcome as the denominator

The prevalence of diabetic retinopathy and nephropathy across phenotypes was estimated and displayed with 95% confidence intervals (Fig. 4). Percentages were calculated using the number of individuals with available data for each outcome as the denominator.

**Fig. 5:**
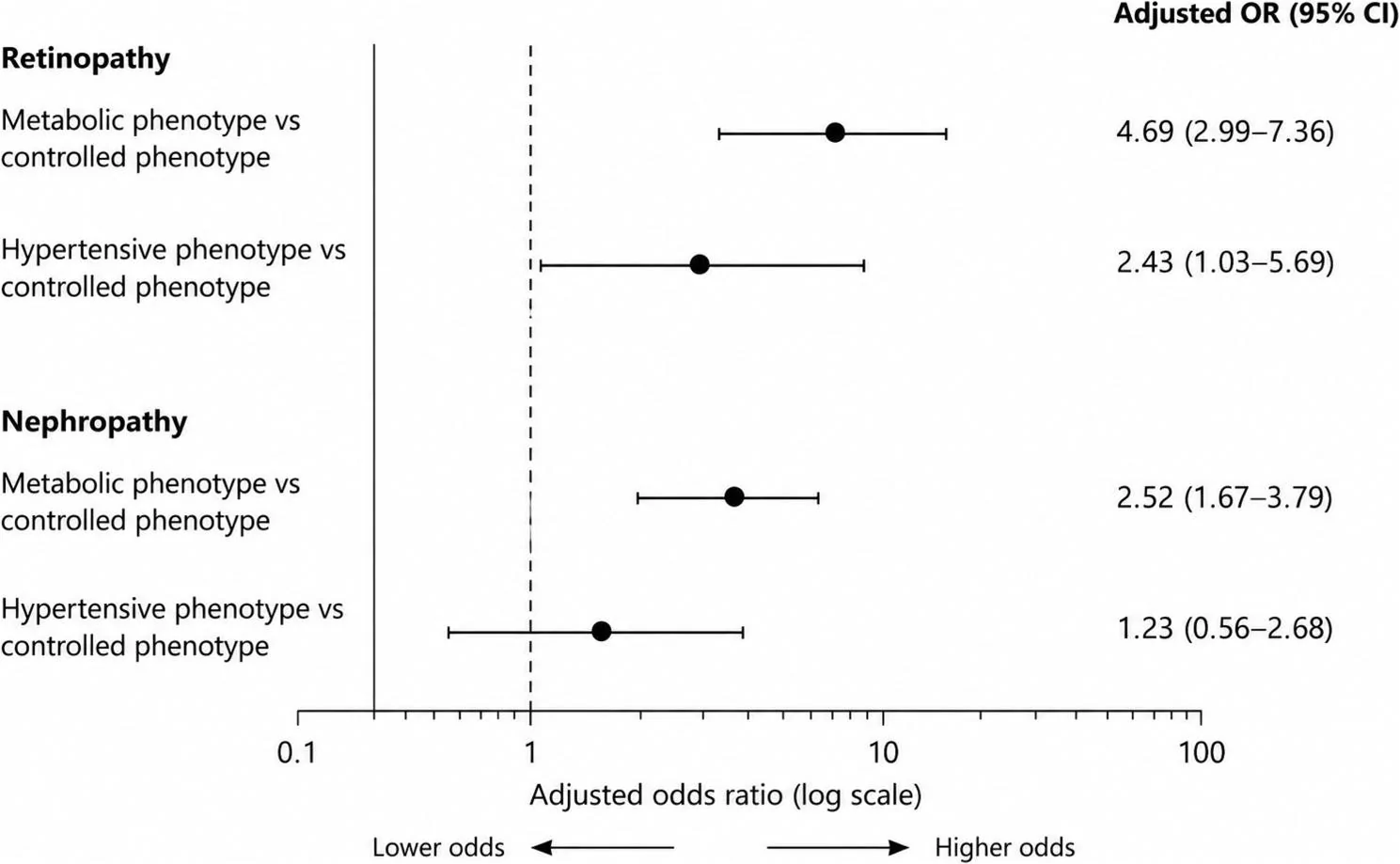
Association between clinical phenotypes and prevalent microvascular complications. Forest plot showing adjusted odds ratios (ORs) and 95% confidence intervals (CIs) for prevalent diabetic retinopathy and prevalent diabetic nephropathy across the identified clinical phenotypes, using the controlled phenotype (Cluster 1) as the reference category. Estimates were obtained from multivariable logistic regression models adjusted for age, sex, and years since diagnosis. The vertical dashed line represents the null value (OR = 1)

Associations between phenotypes and prevalent microvascular complications were assessed using logistic regression models. Crude and adjusted odds ratios (ORs) with 95% confidence intervals (CIs) were estimated for both outcomes, using the controlled phenotype as the reference category. Adjusted models included age, sex, and duration of diabetes (Table 4; Fig. 5). All regression analyses were restricted to individuals assigned to one of the three clinical phenotypes and with available data for the corresponding outcome.

**Table 4:** Multivariable logistic regression analysis of prevalent microvascular complications according to clinical phenotype

Outcome	Model	Comparison	OR (95% CI)	p-value
Retinopathy	Crude	Metabolic phenotype (Cluster 2) vs controlled phenotype (Cluster 1)	5.39 (3.63–8.01)	<0.001
	Crude	Hypertensive phenotype (Cluster 3) vs controlled phenotype (Cluster 1)	1.49 (0.70–3.19)	0.306
	Adjusted*	Metabolic phenotype (Cluster 2) vs controlled phenotype (Cluster 1)	4.69 (2.99–7.36)	<0.001
	Adjusted*	Hypertensive phenotype (Cluster 3) vs controlled phenotype (Cluster 1)	2.43 (1.03–5.69)	0.042
Nephropathy	Crude	Metabolic phenotype (Cluster 2) vs controlled phenotype (Cluster 1)	2.98 (2.02–4.39)	<0.001
	Crude	Hypertensive phenotype (Cluster 3) vs controlled phenotype (Cluster 1)	1.10 (0.52–2.34)	0.807
	Adjusted*	Metabolic phenotype (Cluster 2) vs controlled phenotype (Cluster 1)	2.52 (1.67–3.79)	<0.001
	Adjusted*	Hypertensive phenotype (Cluster 3) vs controlled phenotype (Cluster 1)	1.23 (0.56–2.68)	0.611

*Adjusted for age, sex, and years since diagnosis. The controlled phenotype (Cluster 1) was used as the reference category. Models were fitted among patients assigned to one of the three clinical phenotypes and with available outcome data

Statistical analyses were performed using IBM SPSS Statistics for Windows, version 29.0 (IBM Corp., Armonk, NY, USA) and Python version 3.11. Cluster analysis and visualisation were conducted using the scikit-learn and matplotlib libraries.

### Model performance and sensitivity analysis

**Fig. 6:**
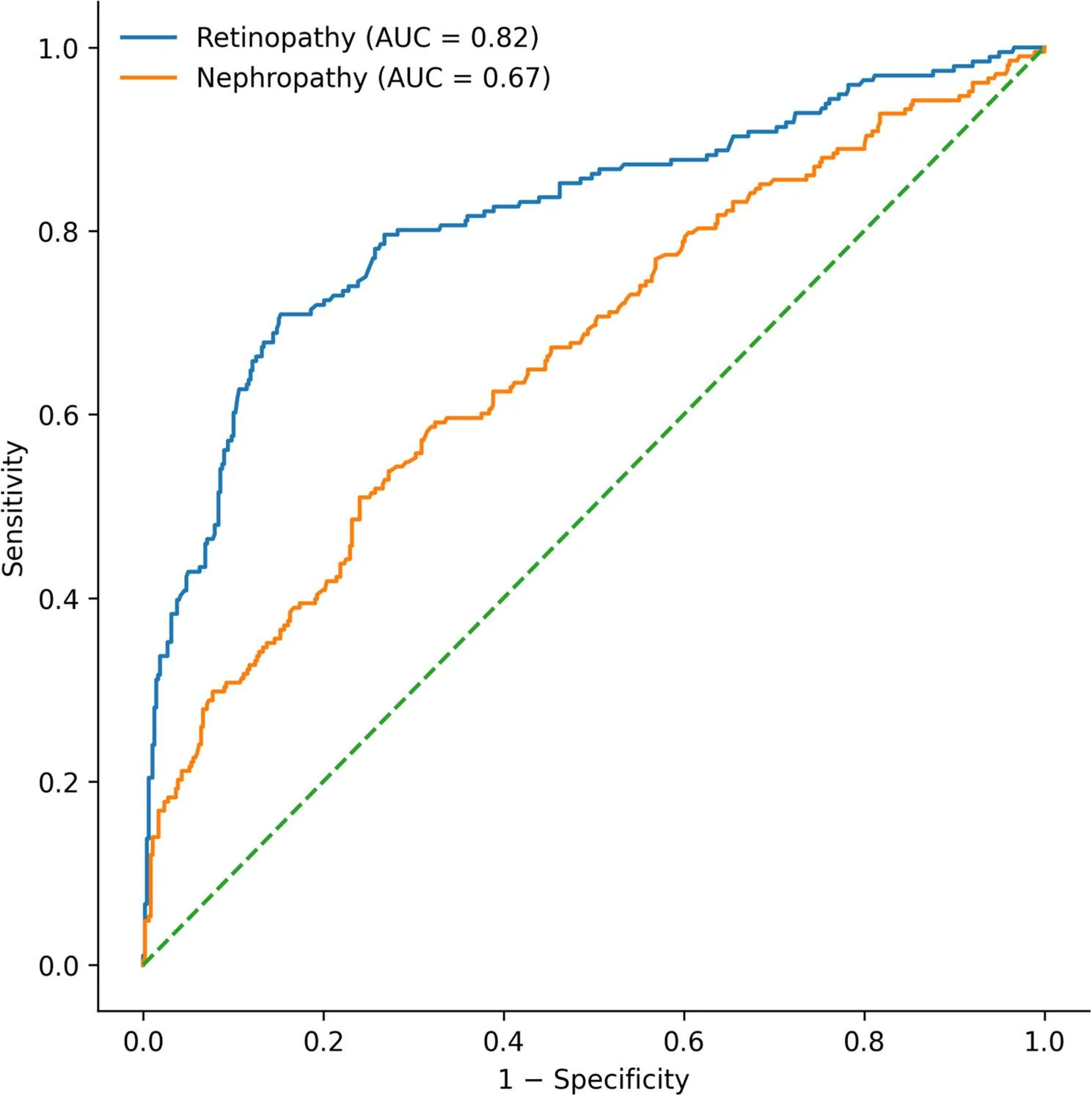
Receiver operating characteristic curves for prevalent microvascular complications. ROC curves showing the discriminatory performance of the multivariable logistic regression models for prevalent diabetic retinopathy and prevalent diabetic nephropathy. The diagonal dashed line represents the line of no discrimination. AUC: area under the receiver operating characteristic curve

Descriptive model performance was assessed in terms of discrimination, calibration, explanatory capacity, and overall model performance. Discrimination was evaluated using the area under the receiver operating characteristic curve (AUC), calibration using the Hosmer–Lemeshow goodness-of-fit test, explanatory capacity using Nagelkerke R², and overall model performance using the Brier score. ROC curves were generated for both prevalent outcomes (Fig. 6).

Sensitivity analyses were performed by repeating the adjusted logistic regression models after exclusion of the hypertensive phenotype because of its smaller sample size. Results are presented in Supplementary Table S1.

### Ethical considerations

The study was conducted using anonymised clinical data obtained from electronic health records and routine clinical assessments. Written informed consent was obtained from all participants prior to inclusion. The study protocol was approved by the Clinical Research Ethics Committee of Cantabria (internal code 2014.151; Act 23/2014). All procedures were conducted in accordance with the Declaration of Helsinki and applicable ethical and data protection regulations.

## Results

### Study population

A total of 742 people with type 2 diabetes mellitus were initially identified from primary care clinical records. After exclusion of 62 individuals due to missing key variables, the final analytical sample comprised 680 individuals (Fig. 1). Phenotype assignment was possible for 674 individuals with complete information for the classification criteria. These individuals were classified into three clinical phenotypes: controlled phenotype (Cluster 1, *n* = 492), metabolic phenotype (Cluster 2, *n* = 146), and hypertensive phenotype (Cluster 3, *n* = 36). Six individuals were not assigned to one of the three phenotypes.

### Descriptive characteristics

Descriptive characteristics differed significantly across phenotypes (Table 1).

Individuals in the controlled phenotype (Cluster 1) were older than those in the hypertensive phenotype (Cluster 3) (70.3 ± 12.9 vs. 65.6 ± 11.3 years; *p* = 0.026). The proportion of male participants was higher in the hypertensive phenotype (72.2%) than in the controlled and metabolic phenotypes (51.0% and 50.7%, respectively; *p* = 0.045). Duration of diabetes was longest in the metabolic phenotype (12.0 ± 5.7 years), followed by the controlled phenotype (9.5 ± 4.1 years) and the hypertensive phenotype (7.9 ± 3.8 years) (*p* < 0.001).

### Cluster characteristics

Distinct clinical profiles were observed across phenotypes (Table 3; Fig. 3). The controlled phenotype (Cluster 1) was characterised by HbA1c within target range and absence of diastolic hypertension. The metabolic phenotype (Cluster 2) was characterised by HbA1c above target, higher BMI, and the longest duration of diabetes. The hypertensive phenotype (Cluster 3) was characterised by a high prevalence of elevated systolic (83.3%) and diastolic (100%) blood pressure levels.

### Principal component analysis

Principal component analysis was used for post-hoc visualisation of the identified phenotypes (Fig. 2). The hypertensive phenotype (Cluster 3) showed visual separation from the remaining phenotypes, whereas the controlled and metabolic phenotypes (Clusters 1 and 2) demonstrated partial overlap, suggesting a gradual clinical continuum between these groups.

### Microvascular complications

The prevalence of microvascular complications differed significantly across phenotypes (Table 2; Fig. 4). Diabetic retinopathy was observed in 58.2% of individuals in the metabolic phenotype and 27.8% in the hypertensive phenotype, compared with 20.5% in the controlled phenotype (*p* < 0.001). Similarly, diabetic nephropathy was more prevalent in the metabolic phenotype (51.1%) than in the controlled phenotype (25.9%) and hypertensive phenotype (27.8%) (*p* < 0.001). Percentages were calculated using the number of individuals with available data for each outcome as the denominator.

### Association between phenotypes and prevalent complications

Multivariable logistic regression analyses are presented in Table 4; Fig. 5. After adjustment for age, sex, and duration of diabetes, the metabolic phenotype (Cluster 2) was associated with higher odds of prevalent retinopathy (OR 4.69, 95% CI 2.99–7.36; *p* < 0.001) and prevalent nephropathy (OR 2.52, 95% CI 1.67–3.79; *p* < 0.001) compared with the controlled phenotype. The hypertensive phenotype (Cluster 3) was associated with prevalent retinopathy (OR 2.43, 95% CI 1.03–5.69; *p* = 0.042), but not with prevalent nephropathy (OR 1.23, 95% CI 0.56–2.68; *p* = 0.611).

### Model performance

Model performance metrics are summarised in Table 5; Fig. 6. The retinopathy model showed higher discrimination (AUC 0.82, 95% CI 0.78–0.86), satisfactory calibration (Hosmer–Lemeshow *p* = 0.71), and moderate explanatory capacity (Nagelkerke R² = 0.23). The nephropathy model showed lower discrimination (AUC 0.67, 95% CI 0.63–0.72), although calibration remained acceptable (Hosmer–Lemeshow *p* = 0.58), suggesting greater heterogeneity in the cross-sectional distribution of prevalent nephropathy.

**Table 5:** Performance of the multivariable models for prevalent microvascular complications

Metric	Retinopathy	Nephropathy
AUC (95% CI)	0.82 (0.78–0.86)	0.67 (0.63–0.72)
Brier score	0.15	0.21
Nagelkerke R²	0.23	0.14
Hosmer–Lemeshow p-value	0.71	0.58
Events, n	196	208
Non-events, n	478	455
Total analysed, n	674	663

The models assessed discrimination for prevalent diabetic retinopathy and prevalent diabetic nephropathy. The Hosmer–Lemeshow test was used to assess model calibration, with p-values >0.05 indicating acceptable fit. Nagelkerke R² reflects the explanatory capacity of the model. The Brier score reflects overall model performance, with lower values indicating better performance. Models were adjusted for age, sex, and years since diagnosis. Analyses were restricted to patients assigned to one of the three clinical phenotypes and with available outcome data; therefore, the total analysed differs between retinopathy and nephropathy

*AUC *Area under the receiver operating characteristic curve

### Sensitivity analysis

Sensitivity analyses excluding the hypertensive phenotype (Cluster 3) yielded consistent results (Supplementary Table S1). The metabolic phenotype (Cluster 2) remained associated with prevalent retinopathy (OR 4.70, 95% CI 3.00–7.37; *p* < 0.001) and prevalent nephropathy (OR 2.36, 95% CI 1.58–3.52; *p* < 0.001).

## Discussion

### Principal findings

In this study, three clinically interpretable phenotypes of type 2 diabetes were identified in a real-world primary care population using a clustering approach based on routinely available clinical variables. These phenotypes showed differentiated clinical profiles and were associated with different patterns of prevalent microvascular complications.

The main finding was that the metabolic phenotype (Cluster 2), characterised by HbA1c above target, higher BMI, and longer duration of diabetes, showed the highest prevalence and higher adjusted odds of both prevalent diabetic retinopathy and prevalent diabetic nephropathy. In contrast, the hypertensive phenotype (Cluster 3), characterised by predominant systolic and diastolic blood pressure elevation, showed a selective association with prevalent retinopathy but not with prevalent nephropathy. The controlled phenotype (Cluster 1) showed the lowest prevalence of both microvascular complications.

### Interpretation of findings

Previous studies have shown that individual clinical factors such as hyperglycaemia, hypertension, obesity, and duration of diabetes are associated with microvascular complications. More recently, clustering approaches have been proposed to better capture the clinical heterogeneity of type 2 diabetes and to identify subgroups with different complication profiles.

The growing interest in population stratification in type 2 diabetes reflects the need to better characterise disease heterogeneity and personalise care. However, much of the earlier work has relied on expert-driven approaches based on predefined criteria, whereas data-driven clustering methods remain comparatively underutilised [6].

Several studies have demonstrated that data-driven clustering can identify clinically meaningful diabetes subgroups with distinct complication profiles. For example, validation studies of the Ahlqvist classification have shown that specific clusters are associated with different renal and retinal outcomes, supporting the role of clustering approaches in describing clinically relevant heterogeneity in diabetes [9]. Additionally, analyses from clinical trial data have shown that distinct subgroups may exhibit differential responses to treatment and varying complication profiles, further supporting the clinical relevance of diabetes phenotyping [15].

Importantly, real-world studies conducted in primary care settings have also supported the feasibility of using routinely collected clinical data for phenotype identification. For instance, Mathur et al. identified clinically meaningful subgroups of people with type 2 diabetes using routinely collected primary care data, demonstrating differences in glycaemic status, treatment initiation, and vascular outcomes across clusters [11].

Similarly, Wang et al. identified distinct clusters of people with type 2 diabetes showing different profiles of microvascular complications, including diabetic nephropathy and retinopathy [10].

Taken together, these findings support the concept that type 2 diabetes is not a homogeneous condition but rather a spectrum of overlapping clinical phenotypes. This is consistent with the patterns observed in our study, where the controlled and metabolic phenotypes partially overlapped in the PCA visualisation, while the hypertensive phenotype showed clearer separation. The terms “metabolic” and “hypertensive” should therefore be interpreted as descriptive labels reflecting the dominant characteristics of the clusters identified in this dataset, rather than as fixed or mutually exclusive pathophysiological subtypes.

Recent work has also highlighted the limitations of clustering approaches, particularly regarding classification uncertainty and the fact that individuals may not fit perfectly into discrete subgroups [16]. This is important for clinical interpretation, because phenotype assignment should be understood as a probabilistic and context-dependent simplification of a continuous disease spectrum rather than an absolute classification.

The partial overlap observed between the controlled and metabolic phenotypes in our PCA plot supports this interpretation. Recent studies have further extended phenotype-based approaches to diabetes and prediabetes populations, showing that data-driven subgroups may differ in microvascular features, diabetes progression, body composition, and response to preventive or therapeutic strategies [17–19].

The higher burden of prevalent retinopathy and nephropathy observed in the metabolic phenotype is biologically plausible, given the role of glycaemic exposure, obesity, and longer diabetes duration in microvascular damage. In our study, the metabolic phenotype may therefore reflect a subgroup with greater cumulative metabolic burden and longer disease exposure. Other studies using metabolic or obesity-related phenotyping approaches have also shown that heterogeneous metabolic profiles may be associated with prevalent type 2 diabetes and diabetes-related metabolic trajectories, supporting the relevance of metabolic clustering for describing cardiometabolic heterogeneity [20, 21]. Evidence also suggests that glycaemic variability and sustained HbA1c elevation are associated with diabetic retinopathy and other microvascular outcomes [22]. In addition, the co-occurrence of diabetic retinopathy and diabetic kidney disease supports the concept of shared microvascular pathways [3].

Duration of diabetes also differed across the identified phenotypes, being longest in the metabolic phenotype, intermediate in the controlled phenotype, and shortest in the hypertensive phenotype. This pattern is clinically relevant because diabetes duration is a well-established marker of cumulative exposure to hyperglycaemia and other metabolic abnormalities. The longer duration observed in the metabolic phenotype, together with HbA1c above target and higher BMI, may help explain the higher burden of prevalent retinopathy and nephropathy observed in this group. This interpretation is consistent with previous evidence linking longer diabetes duration to a greater burden of microvascular complications. Nevertheless, given the cross-sectional design, duration of diabetes should be interpreted as a marker of cumulative disease exposure rather than as evidence of longitudinal progression or prediction of future complications.

The hypertensive phenotype showed a different pattern, with an association with prevalent retinopathy but not with prevalent nephropathy after adjustment. This finding should be interpreted cautiously because of the relatively small size of this cluster. Nevertheless, it may suggest that blood pressure-related profiles do not necessarily overlap completely with metabolic profiles and that different clinical dimensions may contribute to different patterns of microvascular complication burden.

### Clinical implications

These findings may have practical implications for descriptive clinical phenotyping in primary care. The use of routinely collected variables such as age, BMI, HbA1c, blood pressure, and duration of diabetes may help clinicians recognise patterns of clinical complexity among people with type 2 diabetes. In particular, the metabolic phenotype identified in this study was characterised by HbA1c above target, higher BMI, longer duration of diabetes, and a higher prevalence of microvascular complications, suggesting a profile that may warrant careful review of routine complication screening and clinical management.

From a clinical perspective, these phenotypes should be interpreted in relation to the overlapping cardiometabolic profiles commonly seen in primary care. The metabolic phenotype identified in this study shares some features with metabolic syndrome, including higher BMI, HbA1c above target, and greater complication burden. However, it should not be considered equivalent to metabolic syndrome, because the clustering model did not include all classical diagnostic components such as triglycerides, HDL cholesterol, waist circumference, or liver-related markers. Similarly, the hypertensive phenotype should be interpreted as a blood pressure-dominant pattern within the variables included in the clustering model, rather than as a mutually exclusive pathophysiological subtype. These phenotypes may therefore help clinicians recognise dominant patterns of clinical complexity while acknowledging that individual patients may show overlapping metabolic, vascular, renal, and cardiovascular features.

The hypertensive phenotype highlights the importance of blood pressure patterns as part of the overall clinical assessment of people with type 2 diabetes. However, given the cross-sectional design, these findings should not be interpreted as evidence that phenotype membership predicts future complications. Rather, they describe patterns of co-occurrence between clinical profiles and prevalent microvascular complications.

The feasibility of this approach is strengthened by the use of routinely available primary care variables, without the need for complex or costly biomarkers.

Nevertheless, any use of phenotype-based classification to support clinical decision-making, electronic health record tools, or risk stratification would require external validation and longitudinal assessment before implementation.

### Model performance and robustness

The multivariable models showed higher discrimination for prevalent retinopathy and more modest discrimination for prevalent nephropathy. The lower discriminatory performance observed for nephropathy may reflect the more heterogeneous and multifactorial nature of diabetic kidney disease, which involves metabolic, haemodynamic, inflammatory, and genetic mechanisms beyond glycaemic status alone. This is consistent with recent evidence showing that modelling of diabetic kidney disease often requires broader sets of clinical, biochemical, and machine-learning-derived predictors than those included in simple descriptive phenotype models [23].

Sensitivity analyses excluding the hypertensive phenotype supported the robustness of the association between the metabolic phenotype and prevalent microvascular complications. However, these analyses should be interpreted as supportive rather than confirmatory, given the observational and cross-sectional nature of the study.

### Strengths and limitations

This study has several strengths. It was conducted in a real-world primary care population, enhancing the clinical relevance of the findings. The original sampling strategy was population-based and used probability proportional to size sampling across primary care centres. The use of routinely available clinical variables increases the feasibility of applying similar descriptive phenotyping approaches in everyday primary care. In addition, the clustering strategy enabled the identification of clinically interpretable phenotypes, and sensitivity analyses supported the stability of the main findings.

However, some limitations should be acknowledged. First, the cross-sectional analytical design precludes establishing temporal relationships between phenotype assignment and microvascular complications. Therefore, the observed associations should be interpreted as patterns of co-occurrence with prevalent complications rather than evidence of causality, prediction, or incident risk. Longitudinal studies are required to determine whether these phenotypes have prognostic value.

Second, the relatively small size of the hypertensive phenotype may limit the precision of estimates for this group, as reflected by wider confidence intervals. Findings involving this cluster should therefore be interpreted cautiously.

Third, some variables used to define the clusters, particularly HbA1c and blood pressure, are also biologically related to the studied outcomes. As a result, part of the observed associations may reflect shared underlying biological pathways rather than independent cluster effects. Because duration of diabetes was included both in phenotype derivation and as an adjustment variable, adjusted estimates should be interpreted as associations conditional on this component of the phenotype definition.

Conversely, renal markers such as estimated glomerular filtration rate and urinary albumin-to-creatinine ratio were not included in the phenotype derivation because they formed part of the ascertainment and operational definition of prevalent diabetic nephropathy. Including these variables in the clustering procedure or main phenotype-based models would have introduced circularity and may have artificially increased associations or model performance for nephropathy. Future studies may explore alternative phenotyping strategies incorporating renal markers when the objective is to describe broader cardiometabolic or renal phenotypes rather than to assess nephropathy as an outcome.

Fourth, the study relied on routinely collected primary care data, which may be affected by variability in recording practices, data completeness, and clinical coding across clinicians and centres. Although the use of routinely available variables increases the feasibility of descriptive phenotyping in primary care, these data may be incomplete, inconsistently recorded, or influenced by clinical indication. BMI and blood pressure are routinely collected in diabetes care, but selective recording or missingness related to clinical indication cannot be completely ruled out. Therefore, future implementation of phenotype-based approaches in electronic health record systems would require careful data quality assessment, standardised definitions, and external validation. The original sample included 742 individuals, of whom 62 were excluded because of missing blood pressure measurements, incomplete laboratory data, or missing key study variables. Detailed information on these excluded individuals was not available in the analytical dataset, and therefore we could not directly compare included and excluded individuals. Consequently, selection bias related to missing data cannot be completely ruled out, particularly if missingness was associated with clinical characteristics or patterns of routine data recording.

Fifth, this study was conducted in a single health service and region in Spain. External validation in other primary care populations and healthcare systems is needed to assess the generalisability and transportability of the identified phenotypes. In addition, model performance metrics were estimated in the same dataset used for model fitting. Although these metrics were used descriptively to assess discrimination and calibration for prevalent outcomes, the absence of formal internal validation using bootstrap resampling or cross-validation may have led to over-optimistic performance estimates.

Finally, k-means clustering may be sensitive to skewed distributions, scaling choices, and outliers. Although variables were standardised before clustering and the three-cluster solution was supported by clinical interpretability and supplementary clustering diagnostics, future studies should assess the robustness of these phenotypes using internal validation procedures, external validation cohorts, and alternative clustering approaches.

## Conclusions

In conclusion, three clinically interpretable phenotypes of type 2 diabetes were identified in a real-world primary care population and were associated with different patterns of prevalent microvascular complications. The metabolic phenotype showed the highest prevalence and higher adjusted odds of both diabetic retinopathy and nephropathy, while the hypertensive phenotype showed a more selective association with prevalent retinopathy.

These findings suggest that routinely available primary care data can be used to describe meaningful patterns of co-occurrence between clinical phenotypes of type 2 diabetes and microvascular complications. Given the cross-sectional design, these results should not be interpreted as causal or predictive. Longitudinal studies and external validation are needed before these phenotypes can be considered for clinical risk stratification or decision support in routine primary care. 

## Supplementary Information

Supplementary Material 1.

Supplementary Material 2.

Supplementary Material 3.

## Data Availability

The datasets analysed during the current study are available from the corresponding author on reasonable request, subject to applicable data protection regulations.
