# Supplementary material for: Clinical phenotypes of type 2 diabetes and their association with microvascular complications in primary care: a cluster analysis
Source: BMC Prim Care. 2026 Jul 6;27:342. doi: 10.1186/s12875-026-03458-4 (PMC13617723; doi:10.1186/s12875-026-03458-4)
Supplement: Supplementary file 1 [file 12875_2026_3458_MOESM1_ESM.docx]

**Supplementary Figure S1. Elbow and silhouette analysis**

Elbow plot and silhouette analysis used to support the selection of the number of clusters. The elbow method demonstrated an inflection point at k = 3. Although the silhouette coefficient was marginally higher for the two-cluster solution, the three-cluster model was selected because it provided greater clinical interpretability and more meaningful phenotypic differentiation while maintaining acceptable cluster separation.
