# Supplementary material for: Clinical phenotypes of type 2 diabetes and their association with microvascular complications in primary care: a cluster analysis
Source: BMC Prim Care. 2026 Jul 6;27:342. doi: 10.1186/s12875-026-03458-4 (PMC13617723; doi:10.1186/s12875-026-03458-4)
Supplement: Supplementary file 2 [file 12875_2026_3458_MOESM2_ESM.docx]

**Supplementary Table S1. Sensitivity analysis excluding the hypertensive phenotype (Cluster 3)**

| **Outcome** | **Comparison** | **OR (95% CI)** | **p-value** |
| --- | --- | --- | --- |
| Retinopathy | Metabolic phenotype (Cluster 2) vs controlled phenotype (Cluster 1) | 4.70 (3.00–7.37) | <0.001 |
| Nephropathy | Metabolic phenotype (Cluster 2) vs controlled phenotype (Cluster 1) | 2.36 (1.58–3.52) | <0.001 |

**Footnote:**

Odds ratios (ORs) and 95% confidence intervals (CIs) were estimated using multivariable logistic regression models after exclusion of the hypertensive phenotype (Cluster 3). Models were adjusted for age, sex, and years since diagnosis. The controlled phenotype (Cluster 1) was used as the reference category. Analyses were restricted to patients with available outcome data.
