# Supplementary material for: Clinical phenotypes of type 2 diabetes and their association with microvascular complications in primary care: a cluster analysis
Source: BMC Prim Care. 2026 Jul 6;27:342. doi: 10.1186/s12875-026-03458-4 (PMC13617723; doi:10.1186/s12875-026-03458-4)
Supplement: Supplementary file 3 [file 12875_2026_3458_MOESM3_ESM.docx]

**Supplementary Table S2. Availability of key variables in the final analytical sample**

| **Variable** | **Analytical sample, n = 680** | **Available/valid data, n (%)** | **Missing/invalid data, n (%)** |
| --- | --- | --- | --- |
| Age | 680 | 680 (100.0%) | 0 (0.0%) |
| Sex | 680 | 680 (100.0%) | 0 (0.0%) |
| Years since diagnosis | 680 | 680 (100.0%) | 0 (0.0%) |
| BMI | 680 | 677 (99.6%) | 3 (0.4%) |
| HbA1c | 680 | 677 (99.6%) | 3 (0.4%) |
| Systolic blood pressure | 680 | 677 (99.6%) | 3 (0.4%) |
| Diastolic blood pressure | 680 | 677 (99.6%) | 3 (0.4%) |
| Diabetic retinopathy | 680 | 680 (100.0%) | 0 (0.0%) |
| Diabetic nephropathy | 680 | 668 (98.2%) | 12 (1.8%) |

**Footnote:**

Availability of key variables is reported for the final analytical sample of 680 patients. BMI values equal to 0 were considered invalid and treated as missing. Six patients had incomplete information for at least one variable required for phenotype assignment and were therefore not assigned to one of the three clinical phenotypes. **Phenotype-specific analyses were restricted to patients assigned to one of the three clinical phenotypes and with available outcome data.** Detailed information on the 62 patients excluded before construction of the analytical dataset was not available; therefore, a direct comparison between included and excluded individuals could not be performed.
